# 47D11 Antibody-Engineered Exosomes for Targeted Delivery of Remdesivir in Patients with COVID-19: Dream or Principle? (A Critical Editorial Study)

**DOI:** 10.5152/eurasianjmed.2022.21116

**Published:** 2022-10-01

**Authors:** Nahid Daneshi, Abdolreza Esmaeilzadeh, Nazila Bahmaie

**Affiliations:** 1 Zanjan University of Medical Sciences (ZUMS) Faculty of Medicine, Zanjan, Iran; 2 Department of Immunology, Zanjan University of Medical Sciences (ZUMS) Faculty of Medicine, Zanjan, Iran; 3 Immunotherapy Research & Technology Group, Zanjan University of Medical Sciences, Zanjan (ZUMS), Iran; 4 Department of Medical Biology, Ankara Yıldırım Beyazıt University Faculty of Medicine, Ankara, Turkey; 5 Network of Immunity in Infection, Malignancy and Autoimmunity (NIIMA), Universal Scientific Education and Research, Network (USERN), Tehran, Iran

**Keywords:** COVID-19, exosomes, mesenchymal stem cells, regenerative medicine, remdesivir, targeted therapy.

## Abstract

Along with the high transmission rate of coronavirus disease 2019 infection in the last few months, the morbidity and mortality rate of coronavirus disease 2019 has been increased among critically-ill patients, especially the elderly or the ones with immunodeficiencies. So, there is an urgent need to develop more effective therapeutic agents through immunopathophysiological and immunotherapeutic-based strategies for these patients. Here, we hypothesize that mixing S1b-RBD-expressing mesenchymal stem cell-derived exosomes (which have been previously enriched with Remdesivir) with 47D11 antibody, can promisingly guarantee effective transferring of those targeted exosomes to the targeted microenvironment of coronavirus disease 2019 infection. In addition, it can induce their immunomodulatory properties, and anti-viral features, refraining from entrance of severe acute respiratory syndrome-related coronavirus-2 to angiotensin-converting enzyme 2-expressing cells.

In December 2019, an unknown source of pneumonia (as one of the most life-threatening, and a highly contagious viral disease) appeared in the Huanan seafood wholesale market of Wuhan, Hubei province, Wuhan, China. Later, it was renamed as severe acute respiratory syndrome-related coronavirus-2 (SARS-CoV-2) or coronavirus disease 2019 (COVID-19) by the International Committee on Taxonomy of Viruses (ICTV). In the past few months, COVID-19 socioeconomically has attracted worldwide attention with a higher transmission and mortality rates in comparison with other members of this viral family (like Severe Acute Respiratory Syndrome (SARS) and Middle East Respiratory Syndrome (MERS)).^1^

The constantly-growing population of confirmed cases and dead people, the long time needed for processes related to vaccine production, and the inefficiency of current supportive strategies are addressed as problematic challenges of COVID-19, being accounted for unsuccessfulness in COVID-19 treatment. Additionally, inadequacy of therapeutic approaches for all hospitalized patients or those who are ruled out as isolated persons or complied with quarantine situation, and having no definitely Food and Drug Administration (FDA)-approved anti-viral therapeutics all make health managers and basic medical researchers set a contribution and a borderless collaboration in order to reduce the severity of the disease, especially among asymptomatic carriers in the low-income countries. Evidence-based genomic analysis on viral phylogenic relationships suggested that SARS-CoV-2, as a non-segmented, single-stranded, and positive-sense RNA (ssRNA) virus, has identical subgenus and homology of more than 95% to SARS virus, using the same receptor named angiotensin-converting enzyme 2 (ACE2). Structurally, subunits of a full-length spike protein (S1 and S2), as viral transmembrane glycoproteins, are responsible for viral attachment to the host receptors through mediation in the fusion of the viral and cellular membranes, subsequently leading to induction of immune responses. It has been postulated that remarkable affinity of receptor-binding domain (RBD) fragment in S1 subunit for binding to ACE2 significantly increases transmission rate of SARS-CoV-2.^1,2^

On the one hand, such point, lead scientists toward a comprehensive understanding of cellular and molecular mechanisms involved in the immunopathophysiology of COVID-19 infection and microenvironment at the laboratory benches in order to reach to more effective therapeutics at the bedside. On the other hand, it has been demonstrated that the exponential growth of immune-based approaches like “polyclonal antibodies” in the clinical trials has facilitated more accurate and efficacious immunotherapeutic purposes and a revolutionary era for the treatment of immune-mediated disorders. Results from several recently-conducted studies have shown that there are specific humanized polyclonal antibodies against SARS-RBD, depicting cross-neutralizing properties through targeting COVID-19-RBD (conserved epitope), preventing SARS-CoV-2 from entering into ACE2-expressing cells. Sequentially, as it has been demonstrated that S protein in SARS and COVID-19 reveals 77.5% similarity in amino acid sequences, an IgG human anti-SARS molecule called 47D11 antibody (47D11Ab) can bind to S-protein-expressing cells (S1b-RBD) with an acceptable affinity and can simultaneously prevent VEROE6 cells from being infected with SARS and COVID-19 viruses. Hence, it seems that clinical administration of such polyclonal antibodies can be significantly useful for the development of serological tests for the detection of antibodies against COVID-19 infection and alteration by the reduction in the course of viral infectivity.^3^

As the mechanism for the mentioned antibody is different from receptor binding interference, it will be prioritized to use combinative targeted therapies for palliating escape from host immune responses and reaching an increased clinical efficacy. To be more precise, on the one side, remdesivir (REM) (manufactured and distributed by Gilead Sciences, Inc., Foster City, Calif, USA; trade name: Veklury) has been unspecifically considered as the only FDA-approved drug (anti-Ebola drug) with the ability of RNA-dependent RNA polymerase inhibition.^4^

Here, the existence of uncontrolled systematic inflammatory responses in patients with COVID-19 necessitates the utilization of more novel and efficient methods for qualified drug delivery-based approaches in regenerative medicine of infectious diseases, on the other side. Hence, we can imply to immune cell-based therapies like mesenchymal stem cells (MSCs)-based therapies, which have been reported as one of the most efficacious and extensively-used strategies in the phase II and III of pre-clinical trials and clinical studies for a wide array of immune-mediated disorders. Biologically, MSCs are self-renewing, and multipotent stromal cells. They show immunomodulatory and immunosuppressive properties like migration to the damaged tissues and increasing survival rate of damaged cells.^5^ Despite the aforementioned advantages for clinical administration of MSCs, there are still untackled obstacles related to the clinical usage of transplanted MSCs (such as the determination of the most efficient rout and dosage of administration, the possibility for immune-mediated rejections, and senescence-induced genomic instability). These points highlight the importance of the main regeneration activity of stem cells which is indirect and through paracrine manner, developing cell-free treatment (CFT) strategies. Cell-free treatment strategies including exosomes (Exs) can open a promising window for the elimination of shortcomings related to MSC-based therapies, such as bi-functional phenomena, diversion of cell rejection, as well as cell retention. So, it can be speculated that Exs can be a practically and clinically-useful particle as a drug delivery-based method for treatment of COVID-19.^6^

Totally, due to the high spread of COVID-19, and the lack of definitive treatment available for all patients, here we suggest a novel combinative therapeutic approach based on CFT, including targeted therapy with monoclonal antibodies, and delivery of antiviral therapeutics with REM. In this hypothesis-based study, collection, washing, dissolving, determination of morphology, and expression of S1b-RBD-expressing MSC-derived EXs should be conducted. Then, if REM is loaded in isolated S1b-RBD expressing MSC-derived EXs, and then mixed with 47D11Ab overnight, we can promisingly expect that those targeted EXs bind to 47D11Ab, forming a triplicate complex named 47D11Ab-EXs/REM (Figure 1). Needless to say that how important an improved ELISA would be for the determination of optimal proportion of 47D11Ab. Hence, it is of high prominence to evaluate a series of different concentrations of REM, which are incubated with 47D11Ab, and define the most efficient concentration for complex formation. This formation can effectively release REM into the target cells that are involved in the microenvironment of COVID-19 infection. This binding, can show anti-viral effects, as well as inducing immunomodulatory properties of MSCs-derived EXs through prevention from entrance of SARS-CoV-2 to ACE2-expressing cells. We believe that this approach can increase the efficacy and specificity of EXs, mitigate the risk of viral escape from host immune responses, and reduce infectivity by inhibiting the fusion of viral and cellular membranes. It is worth-mentioning that our newly-introduced and hypothetic approach can act as an effective treatment with minimized side effects for patients with COVID-19 (especially for critically-ill patients, and patients with COVID-19 being co-infected with SARS).

Although the merits of this approach have been said earlier, there are still some obstacles that should be tackled. In the case of polyclonal antibody, the specificity of this approach would decrease for SARS-infected people or the ones co-infected with COVID-19 infection. However, in societies lacking reported cases of SARS infection, we would have reasonable specificity and sensitivity for this therapeutic method. Other ambiguities related to this approach are the determination of optimal dose, route, and frequency of administration of EXs for that triplicate structure, short-term, and/or long-term side effects. Despite the necessity of evaluating this method in animal-based and human-based clinical trials, the urgent need for controlling the exacerbation of COVID-19 outbreak can abstain some of these challenges up.

## Figures and Tables

**Figure 1.**
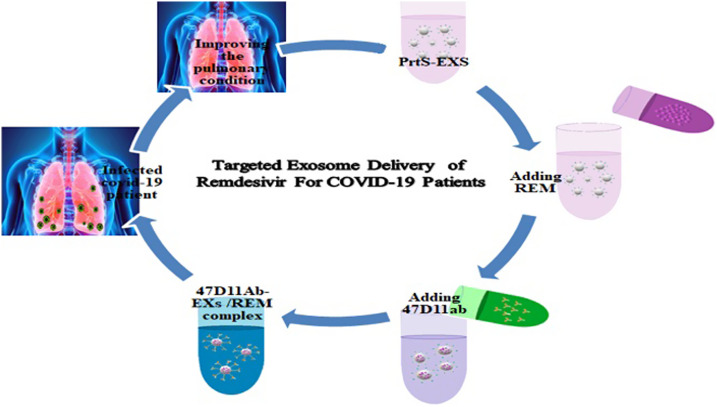
Hypothesis for targeted exosome delivery of remdesivir in COVID-19 patients (created by Esmaeilzadeh et al). PrtS-EXs, exosomes from protein-S-positive mesenchymal stem cells; REM, remdesivir; 47D11ab, 47D11 antibody; 47D11Ab-EXs/REM complex, the complex containing encapsulated remdesivir in protein-S-positive exosome and 47D11 antibody.
